# Long-Term Temporal Trends of *Nosema* spp. Infection Prevalence in Northeast Germany: Continuous Spread of *Nosema ceranae*, an Emerging Pathogen of Honey Bees (*Apis mellifera*), but No General Replacement of *Nosema apis*

**DOI:** 10.3389/fcimb.2017.00301

**Published:** 2017-07-06

**Authors:** Sebastian Gisder, Vivian Schüler, Lennart L. Horchler, Detlef Groth, Elke Genersch

**Affiliations:** ^1^Department of Molecular Microbiology and Bee Diseases, Institute for Bee ResearchHohen Neuendorf, Germany; ^2^Institute of Biochemistry and Biology, University of PotsdamPotsdam-Golm, Germany; ^3^Institut für Mikrobiologie und Tierseuchen, Freie Universität Berlin, Fachbereich VeterinärmedizinBerlin, Germany

**Keywords:** honey bee, *Apis mellifera*, *Nosema* spp., epidemiology, replacement

## Abstract

The Western honey bee (*Apis mellifera*) is widely used as commercial pollinator in worldwide agriculture and, therefore, plays an important role in global food security. Among the parasites and pathogens threatening health and survival of honey bees are two species of microsporidia, *Nosema apis* and *Nosema ceranae. Nosema ceranae* is considered an emerging pathogen of the Western honey bee. Reports on the spread of *N. ceranae* suggested that this presumably highly virulent species is replacing its more benign congener *N. apis* in the global *A. mellifera* population. We here present a 12 year longitudinal cohort study on the prevalence of *N. apis* and *N. ceranae* in Northeast Germany. Between 2005 and 2016, a cohort of about 230 honey bee colonies originating from 23 apiaries was sampled twice a year (spring and autumn) resulting in a total of 5,600 bee samples which were subjected to microscopic and molecular analysis for determining the presence of infections with *N. apis* or/and *N. ceranae*. Throughout the entire study period, both *N. apis*- and *N. ceranae*-infections could be diagnosed within the cohort. Logistic regression analysis of the prevalence data demonstrated a significant increase of *N. ceranae*-infections over the last 12 years, both in autumn (reflecting the development during the summer) and in spring (reflecting the development over winter) samples. Cell culture experiments confirmed that *N. ceranae* has a higher proliferative potential than *N. apis* at 27° and 33°C potentially explaining the increase in *N. ceranae* prevalence during summer. In autumn, characterized by generally low infection prevalence, this increase was accompanied by a significant decrease in *N. apis*-infection prevalence. In contrast, in spring, the season with a higher prevalence of infection, no significant decrease of *N. apis* infections despite a significant increase in *N. ceranae* infections could be observed. Therefore, our data do not support a general advantage of *N. ceranae* over *N. apis* and an overall replacement of *N. apis* by *N. ceranae* in the studied honey bee population.

## Introduction

The Western honey bee *Apis mellifera* is a valuable generalist pollinator for many flowering plants in both natural and agricultural ecosystems. In agriculture, commercial pollination of crop plants, that depend on insect pollination for fruit set and seed production, is provided mostly by managed *A. mellifera* colonies which can, therefore, be regarded as productive livestock. The cultivation of pollinator-dependent crops is expanding all over the world; hence, there is an increasing demand for insect pollination in worldwide agriculture (Aizen et al., 2008, 2009; Aizen and Harder, 2009). Although, this demand is partially met by a globally increasing number of managed honey bee colonies (Aizen et al., 2008, 2009; Moritz and Erler, 2016), increasing problems with honey bee health resulting in severe honey bee colony losses pose a serious threat to human food security. Research of the last decade has identified a multitude of factors like pathogens, pesticides, and abiotic stressors being associated with unusually high and inexplicable losses of honey bee colonies (Genersch, 2010; Ratnieks and Carreck, 2010; Cornman et al., 2012; Pettis et al., 2013; Goulson et al., 2015). Among the pathogens studied and discussed in this context are two microsporidian parasites, *Nosema apis* (*N. apis*) and *N. ceranae*, (Cox-Foster et al., 2007; Higes et al., 2008; Genersch, 2010) which infect adult honey bees (Bailey, 1955).

Microsporidia are highly specialized, spore-forming fungi which are optimally adapted to an obligate intracellular parasitic life style (Keeling and Fast, 2002). Outside of host cells, microsporidia exist as metabolically inactive, infective spores. For *N. apis* and *N. ceranae*, the infection process starts with the ingestion of infective spores by an adult honey bee. The spores germinate in the midgut thereby extruding the polar tube. If the polar tube pierces a host cell, the sporoplasm is injected into the cell through the polar tube (Bigliardi and Sacchi, 2001; Franzen, 2005). Following the injection of the sporoplasm, it takes about 96 h until the first environmental spores are produced by an infected cell (Gisder et al., 2011). The spores are released into the gut lumen through cell lysis and leave the body of the infected host by defecation (Bailey, 1955; Bailey and Ball, 1991). Heavy *Nosema* spp.-infections of adult honey bees may result in dysentery (Bailey, 1967). Adult bees suffering from diarrhea will show abnormal defecation behavior, i.e., will defecate inside the hive, resulting in fecal spots on combs and frames. Nest mates cleaning these spots will ingest *Nosema* spp. spores and become infected (Bailey and Ball, 1991). Infections with *Nosema* spp. are widespread in honey bee populations. Most infected honey bees do not develop nosemosis and do not show any obvious symptoms like dysentery but may have an increased foraging or flight activity (Woyciechowski and Kozlowski, 1998; Dussaubat et al., 2013) despite impaired orientation and homing skills (Kralj and Fuchs, 2010; Wolf et al., 2014) and may have a suppressed immune system (Antunez et al., 2009; Chaimanee et al., 2012), as well as a reduced life span (Wang and Moeller, 1970; Malone and Giacon, 1996; Fries, 2010).

*Nosema apis*-infections in honey bees have been studied intensively over the last 100 years and there is little debate on the rather low impact of this parasite on *A. mellifera* colonies (Bailey and Ball, 1991). However, the impact of *N. ceranae*-infections on colony health and survival is still controversially discussed (Higes et al., 2008; Genersch et al., 2010; Gisder et al., 2010; Guzman-Novoa et al., 2011; Stevanovic et al., 2011; Fernández et al., 2012). The emerging picture is that *N. ceranae* might cause colony death in warmer climates like Southern Europe (Higes et al., 2007, 2008, 2009; Martin-Hernandez et al., 2007; Botías et al., 2013; Cepero et al., 2014) whereas colony losses in Northern Europe or the Americas could not be associated with *N. ceranae* so far (Invernizzi et al., 2009; Genersch et al., 2010; Gisder et al., 2010; Williams et al., 2010; Guzman-Novoa et al., 2011) suggesting a climatic influence on *N. ceranae* virulence (Gisder et al., 2010) or differences in *N. ceranae* susceptibility between regionally predominating *A. mellifera* subspecies (Fontbonne et al., 2013; Huang et al., 2015).

Initially it was thought that *N. apis* is specific for the Western honey bee *A. mellifera* (Zander, 1909), while its congener *N. ceranae* was described as a microsporidian parasite of the Eastern honey bee *A. cerana* (Fries et al., 1996), a native of South- and Southeast Asia. Although, experimental infection showed from the very beginning that *N. ceranae* can also successfully infect *A. mellifera* (Fries, 1997), it took nearly a decade until the first natural infections of *A. mellifera* colonies with *N. ceranae* were reported (Higes et al., 2006; Huang et al., 2007). It soon became evident that *N. ceranae* was not only much more widespread than expected in the global *A. mellifera* populations but that is was even the predominant species in many regions (Klee et al., 2007; Chen et al., 2008; Williams et al., 2008; Invernizzi et al., 2009; Chen and Huang, 2010; Yoshiyama and Kimura, 2011; Copley et al., 2012). Based on this epidemiological evidence it was suggested that *N. ceranae* is replacing *N. apis* in the honey bee populations worldwide. This process is thought to be driven by an asymmetric within-host competition between *N. apis* and *N. ceranae* favoring the spread of *N. ceranae* (Williams et al., 2014; Natsopoulou et al., 2015) although not all studies observed interspecific competition between *N. apis* and its congener *N. ceranae* (Forsgren and Fries, 2010; Milbrath et al., 2015).

However, a pan-European study on the prevalence of *N. apis* and *N. ceranae* reported that in South-European countries, such as Italy and Greece, *N. ceranae* had indeed practically replaced *N. apis* while this was not observed in Northern Europe (Ireland, Sweden, Norway, and Germany) (Klee et al., 2007). These data pointed to climatic factors differentially influencing assertiveness, establishment, spread, and, hence, prevalence of *N. apis* and *N. ceranae*. Experimental evidence exists showing that *N. ceranae* spores, but not *N. apis* spores, nearly lose their ability to germinate and, hence, their infectivity when exposed to temperatures close to or below freezing (Fenoy et al., 2009; Fries, 2010; Gisder et al., 2010). In addition, experimental infection of adult bees showed proliferation of *N. ceranae*—but not of *N. apis*—to be unaffected by temperatures above 33°C (Martin-Hernandez et al., 2009). These data strongly argue for an advantage of *N. ceranae* over *N. apis* in warmer climates. In contrast, the cold-sensitivity of *N. ceranae* spores might slow down the replacement process in colder climates (Gisder et al., 2010), a hypothesis that could recently be substantiated by mathematical modeling of the replacement process when taking into account the parameters warmer and colder climate (Natsopoulou et al., 2015). However, long term epidemiological data on *Nosema* spp. prevalence allowing the observation of the spread of the emerging pathogen *N. ceranae* and evaluating the proposed process of replacement of *N. apis* by *N. ceranae* in a given honey bee population have been lacking so far. To fill this gap, we here present our results of a 12 year cohort study on the prevalence of *N. apis* and *N. ceranae* in Northeast Germany conducted on a cohort of about 230 honey bee colonies. The duration of the study, and the size of the cohort enabled us to statistically analyse the long term temporal trends in prevalence of *N. apis*- and *N. ceranae*-infections in the study area. We also show data from laboratory experiments substantiating our epidemiological data. We provide evidence that the continuous spread of *N. ceranae* and continuously increasing levels of *N. ceranae*-infection prevalence at population level not necessarily result in the replacement of *N. apis*.

## Material and methods

### Bee samples, field survey and molecular differentiation of *N. apis* and *N. ceranae*

**Figure 1:**
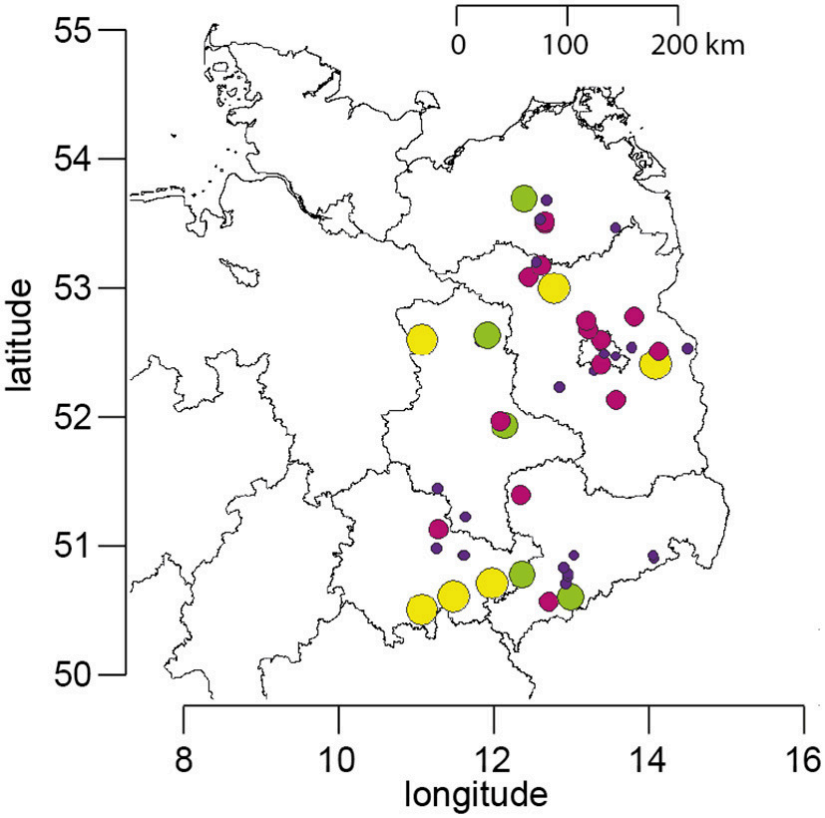
Map of Northeast Germany showing the location of the apiaries which participated in the study. The size and color of the circles represent the number of years for which data are available for each apiary (yellow, 12 years; green, 9–11 years; purple, 5–8 years; blue, 1–4 years).

The data set on *Nosema* spp. prevalence comprises data from spring 2005 to autumn 2016, which were collected in the course of a 5 year longitudinal cohort-study on *Nosema* spp. epidemiology (Gisder et al., 2010) and of the still ongoing “German Bee Monitoring Project” (Genersch et al., 2010). About 23 apiaries located in Northeast-Germany (Figure 1) participated in the projects with 10 colonies (“monitoring colonies”) each. Monitoring colonies that collapsed during the study were replaced by colonies from the same apiary, if available by a nucleus colony made from the collapsed colony in the previous year. This procedure ensured that each apiary always contributed 10 monitoring colonies throughout the study period. Due to the long duration of the study, some fluctuation of participating apiaries could not be avoided. However, nearly half of the apiaries (11 of ~23) participated for more than 9 years and six of them even for the entire duration of the study, i.e., 12 years; at least 20 bee keepers provided samples over a time period of consecutive 5–11 years (Figure 1). When an apiary dropped out, a similar apiary in terms of size, bee race, landscape, region, and history of losses and diseases was chosen as replacement and included in the study as soon as possible. This resulted in an annual mean of 22.67 ± 1.72 (mean ± *SD*) apiaries participating in spring and 24.0 ± 2.83 (mean ± *SD*) apiaries participating in autumn. All monitoring colonies were sampled twice a year, in spring and in autumn, resulting in a total of 5,600 honeybee samples collected and analyzed from the participating apiaries over the 12 year study period (Table 1).

**Table 1:** Prevalence of colonies infected with *N. apis* only (*N. apis*) or *N. ceranae* only (*N. ceranae*) or with *N. apis* and *N. ceranae* (co-infection) from spring 2005 to autumn 2016.

		**Total number of analyzed colonies**	**Infected Colonies [infection categories]**					
			***N. apis***		***N. ceranae***		**co-infection**	
			***n***	**%**	***n***	**%**	***n***	**%**
Spring	2005	220	37	16.8	9	4.1	6	2.7
Autumn	2005	237	19	8.0	10	4.2	1	0.4
Spring	2006	238	43	18.1	10	4.2	14	5.9
Autumn	2006	226	15	6.6	3	1.3	2	0.9
Spring	2007	228	7	3.1	34	14.9	10	4.4
Autumn	2007	219	10	4.6	4	1.8	1	0.5
Spring	2008	209	35	16.8	18	8.6	21	10.1
Autumn	2008	210	6	2.9	5	2.4	0	0.0
Spring	2009	210	33	15.7	23	11.0	12	5.7
Autumn	2009	180	9	5.0	7	3.9	0	0.0
Spring	2010	247	27	10.9	25	10.1	4	1.6
Autumn	2010	250	10	4.0	16	6.4	0	0.0
Spring	2011	230	42	18.3	25	10.9	16	7.0
Autumn	2011	255	7	2.8	7	2.8	5	2.0
Spring	2012	252	24	9.5	33	13.1	23	9.1
Autumn	2012	278	6	2.2	19	6.8	0	0.0
Spring	2013	233	15	6.4	30	12.9	12	5.2
Autumn	2013	257	17	6.6	21	8.2	4	1.6
Spring	2014	224	41	18.3	33	14.7	7	3.1
Autumn	2014	261	4	1.5	8	3.1	0	0.0
Spring	2015	198	25	12.6	15	7.6	7	3.5
Autumn	2015	250	5	2.0	3	1.2	0	0.0
Spring	2016	230	43	18.7	21	9.1	4	1.7
Autumn	2016	258	8	3.1	27	10.5	0	0.0

*Given are the total number of analyzed colonies per year and season as well as the numbers (n) and proportions (%) of colonies within each infection category*.

Sampling of bees as well as diagnosis of *N. apis* and *N. ceranae* were performed essentially as already described (Gisder et al., 2010). Briefly, from each apiary, a group of 10 bee colonies [annual mean: 10 ± 0.31 (mean ± *SD*) colonies in spring and 10.01 ± 0.14 (mean ± *SD*) colonies in autumn] was randomly selected at the beginning of the study or when the beekeeper entered the study and designated “monitoring colonies.” From these colonies, bee samples were collected in spring and autumn each year and were stored at −20°C until analysis. Spring samples collected end of March/beginning of April consisted of dead bees fallen onto the bottom board during the winter season (representing the bees that died over winter) to enable sampling of colonies that collapsed during the winter season (October to March) as well as of surviving colonies. Autumn samples collected in late September/beginning of October consisted of live in-hive bees taken from a super above the queen excluder thus ensuring that only the oldest bees (representing the most frequently infected bees) were sampled (Fries et al., 2013). Diagnosis of *Nosema* spp. infections was performed by microscopic examination of 20 homogenized bee abdomens according to the “Manual of Standards for Diagnostics and Vaccines” published by the Office International des Epizooties (OIE), the World Organization for Animal Health (Anonymous, 2008). The moderate sample size is adequate because the experimental unit is the colony (Doull and Cellier, 1961; Doull, 1965). Infection status of the colonies represents detectable levels of infection above 15% with 96% probability of detection (Fries et al., 1984, 2013; Pirk et al., 2013) which can be considered biologically relevant (Higes et al., 2008). For molecular species differentiation, *Nosema* spp.-positive homogenates were processed and analyzed via PCR-RFLP (restriction fragment length polymorphism) as previously described (Gisder et al., 2010). Results were further verified by re-analyzing randomly selected samples via a recently developed differentiation protocol (Gisder and Genersch, 2013) which is based on the detection of species-specific sequence differences in the highly conserved gene coding for the DNA-dependent RNA polymerase II largest subunit. Based on the diagnostic results, four infection categories were defined: Microscopic analysis resulted in the category “*Nosema* spp.” while molecular differentiation allowed for the categories “*N. apis*” (single infection), “*N. ceranae*” (single infection), and “co-infection” (infection with both *N. ceranae* and *N. apis*) (Table 1).

### Purification of *Nosema* spp. spores for *in vitro*-infection

Honey bee colonies of the apiary of the Institute for Bee Research were screened for *Nosema* spp.-infections by microscopic analysis of 20 randomly collected adult bees (see above and Anonymous, 2008). *Nosema* spp.-positive samples were molecularly differentiated as previously described (Gisder and Genersch, 2013) to identify samples either containing only *N. ceranae* or only *N. apis* spores. Purification of *N. apis* or *N. cerane* spores was exclusively performed with freshly sampled bees, because freezing or long-term storage affect spore viability and infection rate (Fenoy et al., 2009; Fries, 2010; Gisder et al., 2010). Midguts were carefully isolated from individual bees by using fresh forceps for each bee. Twenty midguts were pooled in 1.5 ml reaction tubes and spore purification was performed as already described (Gisder et al., 2010).

Viability of the purified spores was checked via *in vitro*-germination. To this end, an aliquot of freshly isolated spores was air-dried onto glass slides for 30 min at room temperature. Germination was triggered by adding 20 μl of 0.1 M sucrose solution buffer directly to the dried spores. Germination process was analyzed under an inverse microscope (VWR, Darmstadt, Germany) at 400x magnification with phase contrast. *Nosema* spp. spores were counted in a hemocytometer (Neubauer-improved, VWR, Darmstadt, Germany) under an inverse microscope (VWR, Darmstadt, Germany) at 100x magnification. Only those spore preparations that were able to germinate under *in vitro* conditions were used for cell culture experiments.

### *In vitro*-infection of cultured IPL-LD-65Y cells

The insect cell line IPL-LD-65Y derived from the gypsy moth *Lymantria dispar* was obtained from the *Deutsche Sammlung von Mikroorganismen und Zellkulturen* (DSMZ, Braunschweig, Germany) and maintained for routine culture as given in the accompanying data sheet. For *in vitro*-infection of cultured IPL-LD-65Y-cells, aliquots of about 5 × 10^7^ spores, purified as described above, were dried in 1.5 ml reaction tubes (Eppendorf, Hamburg, Germany) in a vacuum concentrator (Eppendorf, Hamburg, Germany) for 30 min at 30°C. Subsequently, infection of IPL-LD-65Y cells with germinating spores was performed as previously described (Gisder et al., 2011). Briefly, IPL-LD-65Y cells were infected with freshly isolated *N. apis* or *N. ceranae* spores with a multiplicity of infection (MOI) of 20. Infected cells (100 μl with 2.5 × 10^5^ cells/ml) were seeded in the cavities of six 96-well microtiter plates. *N. apis*- and *N. ceranae*-infected cells were incubated at 21°, 27°, or 33°C. Infected cells were centrifuged on glass slides at the time points 24, 32, 48, 72, and 96 h post initial infection and were subsequently Giemsa-stained as described (Gisder et al., 2011). The number of meronts, sporonts, and mature spores of *N. apis* or *N. ceranae* was counted under an inverse microscope Eclipse Ti-E (Nikon Instruments, Düsseldorf, Germany) at 600x magnification in 10 individual cells for each time point as well as for each temperature and expressed as mean ± *SD*.

### Statistical analysis

For statistically analyzing the seasonality of *Nosema* infections, spring vs. autumn, the Wilcoxon signed rank test was used because the proportions of infected colonies (Table 1) were not normally distributed. In addition, the Spearman rank correlation was determined with R (version 3.2.5, R Development Core Team, 2016) to analyse the relationship between infection categories. The Spearman correlation coefficient determined the strength of the monotonic relationship between season and infection prevalence with effect sizes between 0.10 and 0.29 representing weak correlations, coefficients between 0.30 and 0.49 representing medium correlations, and coefficients of 0.50 or above representing strong correlations.

For each time point, the expected rate of co-infections (E_co−inf_) was calculated as the product of the observed rates of single infections with either *N. apis* (R_apis_) or *N. ceranae* (R_ceranae_): E_co−inf_ = R_apis_^*^ R_ceranae_. Subsequently, the differences between the observed and expected rates of co-infections were calculated for each time point. Because those differences were normally distributed, a one sample *t*-test was used to check if these differences were significantly different to zero.

The statistical analysis of temporal trends was performed using RStudio (version 0.99.489) based on R using version 3.2.5. For visualizing infection prevalence data, dotplots were plotted with R, separately for spring and autumn. Generalized linear models (GLM) were fitted with lme4 (Linear Mixed-Effects Models, version 1.1-12) (Bates et al., 2015) for exploring the data set and visualizing the relationship between the dependent variables (*Nosema* spp.) and the independent variables (year). For statistical analysis of *N. apis* and *N. ceranae* prevalence over the 12 year study period we used mixed-effect binary logistic regressions analysis defining year as fixed factor and apiary as random factor to take into account the lack of independence of data within each apiary. Even after 12 years of sampling, the amount of data is still not sufficient to define colony as random factor to fully acknowledge relative data dependence. The sampling consisted of about 230 individual colonies per season, stratified within apiaries, and the prevalence of *N. apis*-, *N. ceranae*-, or co-infections at the individual level were analyzed with defining “0” if absent or “1” if present in each colony. Odds ratios (ORs) and 95% confidence intervals [CIs] were used to assess the strength of the associations.

For statistical analysis of the counted number of different developmental stages of *Nosema* spp. in infected IPL-LD-65Y cells, individual student's *t*-tests for each time point were performed followed by Benjamini-Hochberg correction (Benjamini and Hochberg, 1995). A *p* < 0.05 was considered significant for the statistical tests.

## Results

### Prevalence and seasonality of *Nosema* spp.-infections

**Figure 2:**
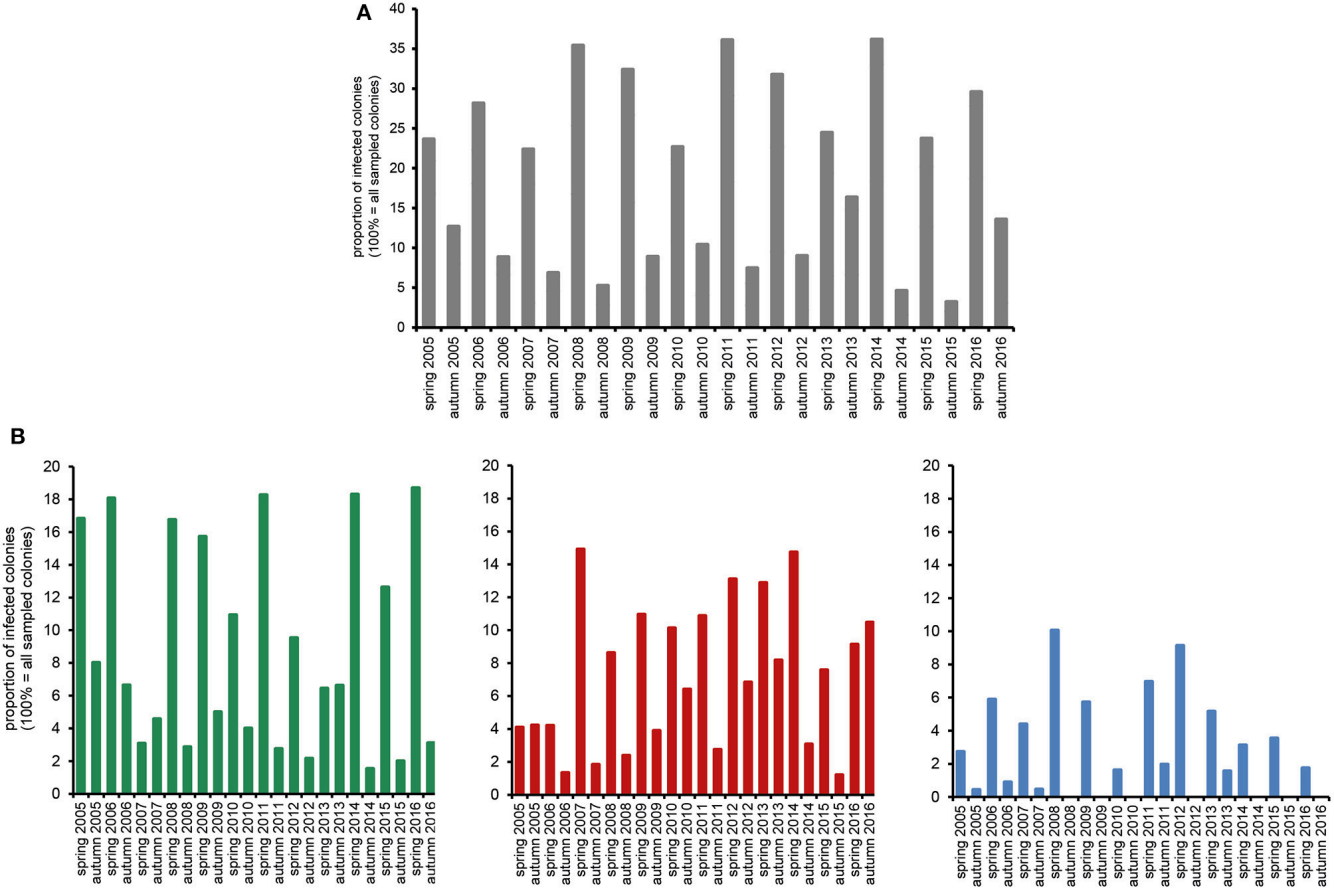
Prevalence in spring and autumn (seasonality) of *Nosema* spp.-infections over the study period from spring 2005 to autumn 2016. **(A)** Proportion of *Nosema* spp.-infections in the study cohort according to microscopic analysis. **(B)** Proportion of *N. apis*- (single infection, green columns), *N. ceranae*- (single infection, red columns), and *N.apis*/*N. ceranae* co-infections (blue columns) as revealed by molecular species differentiation.

The huge data set on *Nosema* spp.-infection prevalence in Northeast Germany, which was generated during the 12 year longitudinal cohort study, provided a unique opportunity for a comprehensive analysis of the spread and success of *Nosema* spp., and especially of *N. ceranae*, in a restricted honey bee population. We first analyzed the seasonality of *Nosema* spp.-infections based on classical microscopic diagnosis without molecular species differentiation. The data revealed a clear and expected (Bailey and Ball, 1991) seasonality of *Nosema* spp.-infections for the whole duration of the study period with spring values being always higher than the autumn values of the same year and autumn values being always lower than the spring values of the following year (Figure 2A).

Molecular species differentiation of all *Nosema* spp.-positive samples enabled analysing the seasonality of *N. apis*-, *N. ceranae*-, and co-infections (Table 1). The same seasonality as already observed for *Nosema* spp.-infections was also evident for *N. apis*-infections over the entire study duration despite for the time point “spring 2007” when less colonies where found infected with *N. apis* than in the preceding autumn 2006 and the following autumn 2007 (Figure 2B). With this exception for “spring 2007,” when only 3.1% of the colonies carried detectable *N. apis*-infections, the proportion of *N. apis*-infected colonies varied between 6.4% (spring 2013), and 18.7% (spring 2016). In autumn, the prevalence of *N. apis*-infected colonies ranged between 1.5% (autumn 2014) and 8.0% (autumn 2005).

For *N. ceranae*-infections, the described seasonality with higher prevalence in spring than in the following autumn and lower prevalence in autumn than in spring next year could be observed from autumn 2006 onward until spring 2016, whereas between spring and autumn 2016 the prevalence of *N. ceranae*-infections did not decrease as expected but instead further increased (from 9.1 to 10.5%; Figure 2B). Spring prevalence from 2007 to 2016 varied for *N. ceranae*-infections between 7.6% (spring 2015) and 14.9% (spring 2007), while autumn prevalence ranged between 1.2% (autumn 2015) and 8.2% (autumn 2013).

The prevalence of colonies co-infected with *N. apis* and *N. ceranae* showed the same seasonal pattern fluctuating between spring (higher prevalence) and autumn (lower to no prevalence). Values for co-infection prevalence ranged between 1.6% (spring 2010) and 10.0% (spring 2008) in spring and between 0.0% (autumn 2008, 2009, 2010, 2012, 2014, 2015) and 2.0% (autumn 2011) in autumn (Figure 2B).

**Figure 3:**
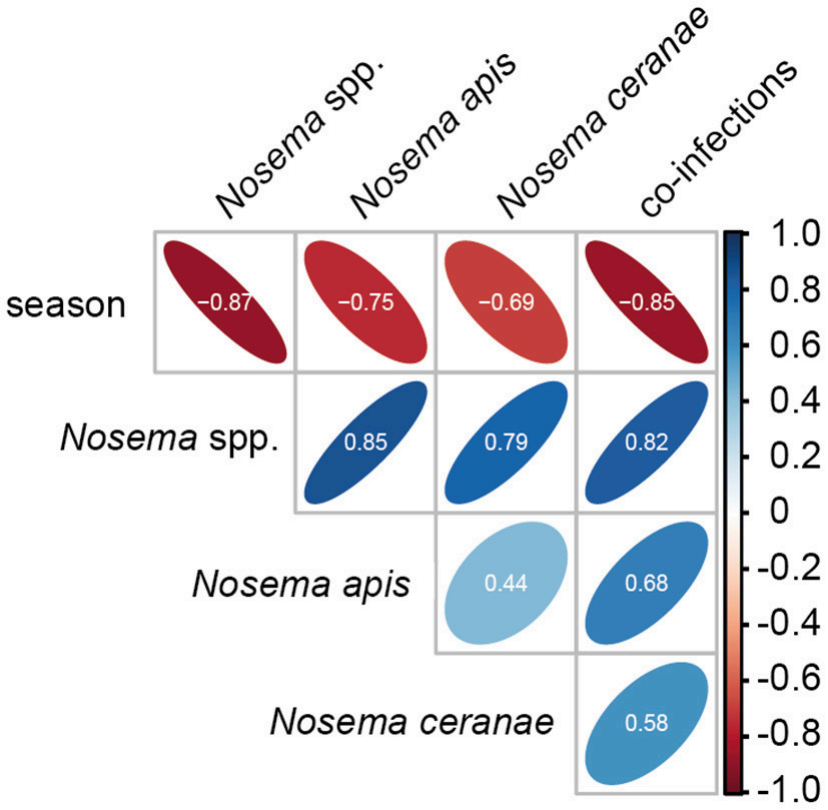
Spearman correlation matrix values showing the pairwise correlation coefficients for the parameters season and the four infection categories (*Nosema* spp.-, *N. apis*-, *N. ceranae*-, and co-infections). Shape and orientation of the ellipses represent the data clouds of the respective correlation coefficients. Positive correlations are illustrated in blue and negative correlations in red. Coefficients (white digits) between |0.10| and |0.29| represent weak associations, coefficients between |0.30| and |0.49| represent medium associations and coefficients of |0.50| or above (beyond) represent strong associations.

Statistical analysis of the seasonality of *Nosema* spp.-, *N. apis*-, *N. ceranae*-, and co-infections using a Mann-Whitney test confirmed the above given, rather descriptive evaluation (for all infection categories, *p* < 0.01). Spearman correlation analysis further substantiated this finding (Figure 3). A strong negative correlation (coefficient values between −0.69 and −0.87) was found between season and all infection categories indicating that in each year and for all four infection categories (*Nosema* spp.-, *N. apis*-, *N. ceranae*-, co-infection) the infection prevalence decreased significantly from spring to autumn (for all infection categories: *p* < 0.01). Medium to strong positive correlations (coefficient values between 0.44 and 0.85) were found between the infection categories implying that all infection categories followed the same prevalence trend. For example, high infection prevalence for *N. apis* correlated with high infection prevalence for *N. ceranae*- or co-infections. This correlation was significant for all infection categories (*p* < 0.05).

**Figure 4:**
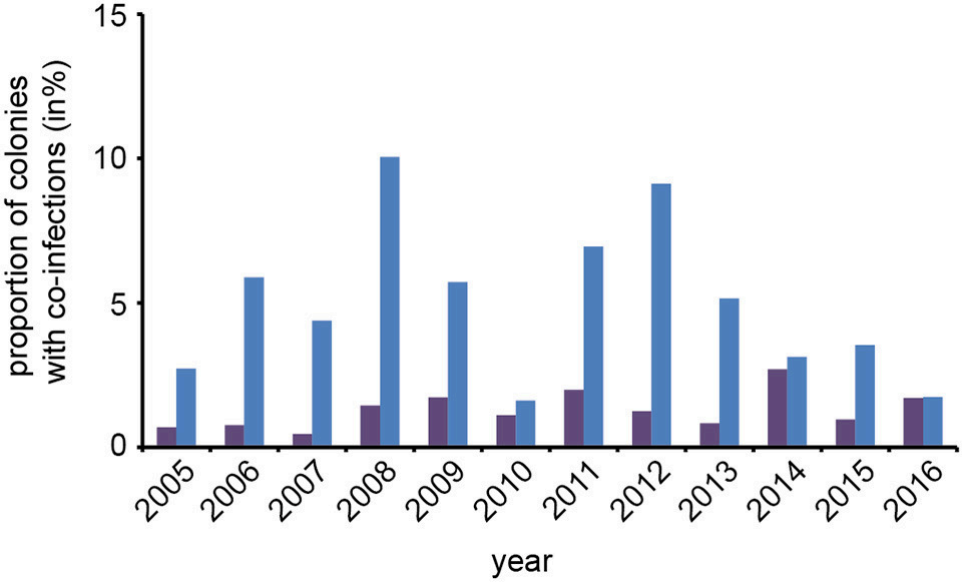
Predicted and observed frequencies of prevalence of colonies being co-infected in spring with *N. apis* and *N. ceranae*. Predicted values for co-infections (violet bars) were calculated from the observed frequencies of *N. apis*- and *N. ceranae*-infected colonies and compared to the observed values for co-infection prevalence (blue bars) for each spring between 2005 and 2016.

An interesting question in regard to co-infections was, whether or not the observed prevalence of co-infections in spring was congruent with the expected prevalence. To answer this question, we first calculated the rate of expected co-infections for each year from the rate of observed *N. apis*- and *N. ceranae*-infections in this season. Comparing these values with the observed frequency of co-infections revealed that over the entire study period, the observed prevalence of co-infections was always significantly {one sample *t*-test; *M* = 0.037, [0.0195, 0.0546], *t*_(23)_ = 4.6389, *p* < 0.01} higher than expected when assuming that the occurrence of co-infections was only influenced by the prevalence of single infections (Figure 4).

### Temporal pattern of *Nosema* spp. prevalence

**Figure 5:**
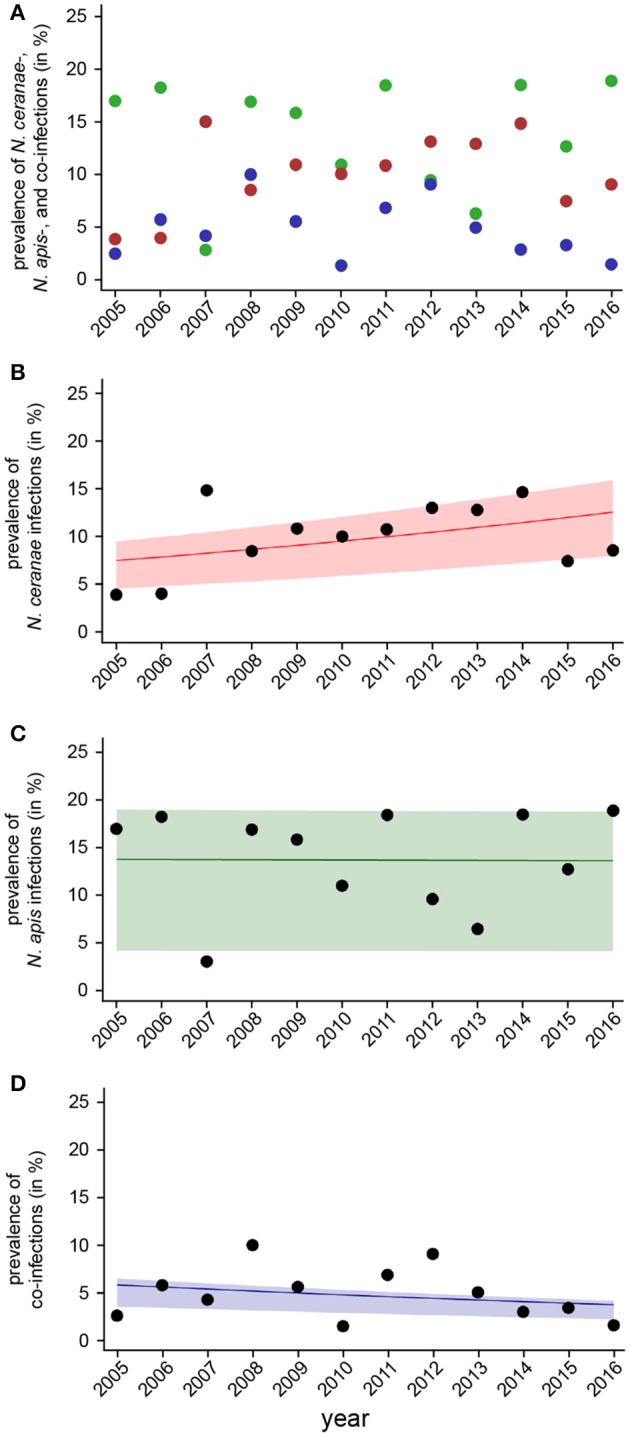
Temporal patterns of prevalence of single infections with *N. ceranae* or *N. apis* and of co-infections detected in spring samples between 2005 and 2016. **(A)** Prevalence data for *N. ceranae-, N. apis*-, and co-infections in spring are plotted against year. **(B)** Data sets for prevalence of *N. ceranae-, N. apis*-, and co-infections in spring were fitted by Linear Mixed-Effects Models to visualize the relationship between the independent variables (year) and the dependent variables *N. ceranae*- **(B)**, *N. apis*- **(C)**, and co-infections **(D)**. Regression lines visualizing trends for *N. ceranae*- (**B**; solid red line), *N. apis*- (**C**; solid green line), and co-infection (**D**; solid blue line) prevalence are shown; ribbons represent the 2nd and 3rd quartile (25–75%) of the predicted data of the model.

**Figure 6:**
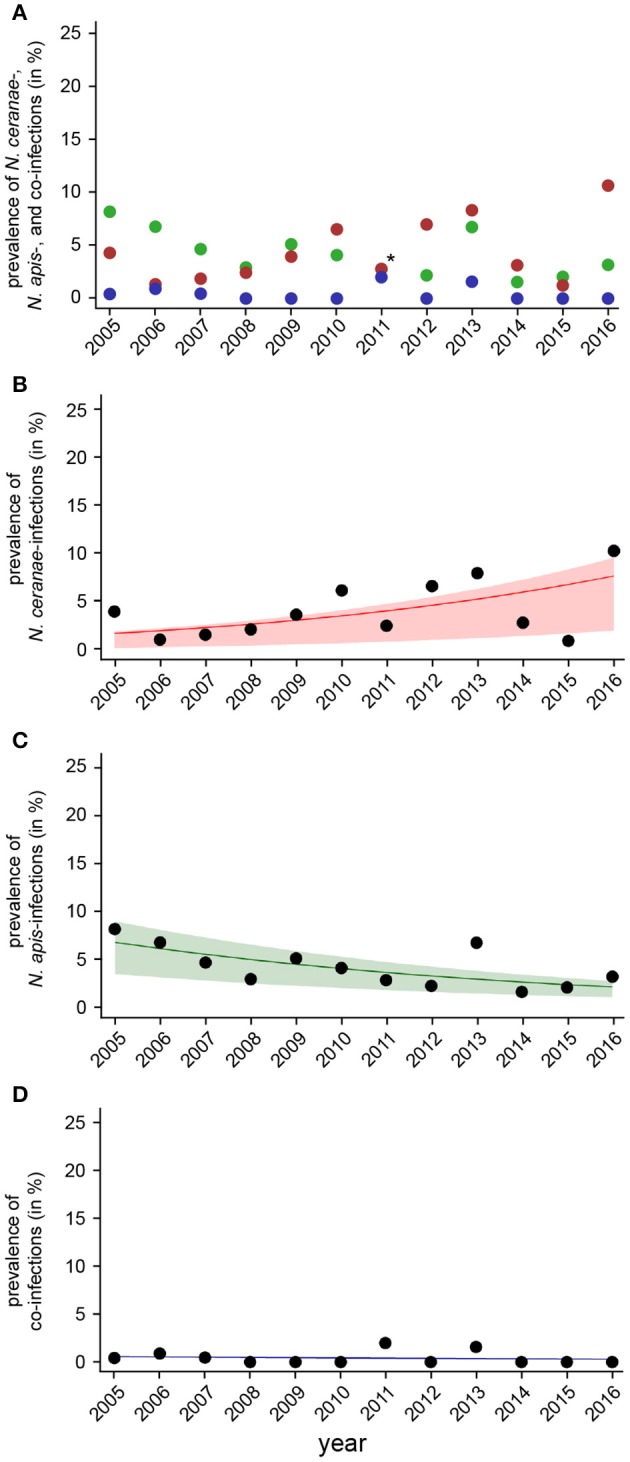
Temporal patterns of prevalence of single infections with *N. ceranae* or *N. apis* and of co-infections detected in autumn samples between 2005 and 2016. **(A)** Prevalence data for *N. ceranae-, N. apis*-, and co-infections in autumn are plotted against year. In autumn 2011, highlighted by an asterisk, the proportions of *N. apis*- and *N. ceranae*-infections were identical, hence, only a red dot is visible. **(B)** Data sets for prevalence of *N. ceranae-, N. apis*-, and co-infections in autumn were fitted by Linear Mixed-Effects Models to visualize the relationship between the independent variables (year) and the dependent variables *N. ceranae*- **(B)**, *N. apis*- **(C)**, and co-infections **(D)**. Regression lines visualizing trends for *N. ceranae*- (**B**, solid red line), *N. apis*- (**C**; solid green line), and co-infection (**D**; solid blue line) prevalence are shown; ribbons represent the 2nd and 3rd quartile (25–75%) of the predicted data of the model.

For evaluating spread and assertiveness of the emerging honey bee pathogen *N. ceranae*, we analyzed the temporal patterns of *N. ceranae*-, *N. apis*-, and co-infections by plotting and statistically analysing the respective values separately for the spring (Figure 5) and autumn (Figure 6) seasons between 2005 and 2016. While the patterns for *N. apis*- and co-infections in spring did not show a consistent trend, the pattern for *N. ceranae*-infection prevalence suggested a continuously increasing trend over the years (Figure 5A). Generalized linear models (GLM) of the prevalence data confirmed this interpretation (Figures 5B–D). Logistic regression analysis (Table 2) demonstrated that the continuous increase in spring prevalence of *N. ceranae*-infections observed over the entire 12 year study period, i.e., between 2005 and 2016, was on average about 5% per year (Odd Ratio: 1.05 [1.01, 1.1]) and was significant (GLM, Likelihood Ratio test of the model, *p* = 0.02) (Figure 5B). This increase, however, was not accompanied by any significant (GLM, Likelihood Ratio test of the model, *p* = 0.95) change in the spring prevalence of *N. apis*-infections (Odd Ratio: 1.0 [0.96, 1.04]) (Figure 5C). Likewise, no significant trends (GLM, Likelihood Ratio test of the model, *p* = 0.17) were observed for co-infections in spring (Odd Ratio: 0.96 [0.9, 1.02]) (Figure 5D).

**Table 2:** Results of the binary logistic regression analysis of prevalence of *N. apis-, N. ceranae*- and co-infections over the 12 year study period (see also Figures 5, 6).

	**Infection categories**	Odd Ratios (CI)^*^	***p*-value**
spring	*N. apis*-infections	0.00 (0.96, 1.04)	0.95
	*N. ceranae*-infections	1.05 (1.01, 1.10)	0.02
	co-infections	0.96 (0.90, 1.02)	0.17
autumn	*N. apis*-infections	0.89 (0.84, 0.95)	0.0003
	*N. ceranae*-infections	1.15 (1.07, 1.25)	0.0003
	co-infections	0.95 (0.81, 1.10)	0.49

* *, 95% confidence interval*.

The dotplot of autumn prevalence of *N. ceranae*-, *N. apis*-, and co-infections (Figure 6A) showed a different pattern with an increasing trend for *N. ceranae*- being accompanied by a decreasing trend for *N. apis*-infections. This finding could be substantiated by GLM-analysis and Likelihood Ratio tests of the models (Table 2, Figures 6B–D). In autumn, the prevalence of *N. ceranae*-infections was significantly (GLM, Likelihood Ratio test of the model, *p* = 0.0003) increasing by an average of about 15 % per year (Odd Ratio: 1.15 [1.07, 1.25]) over the study period (Figure 6B) while at the same time the prevalence of *N. apis*-infections was significantly decreasing by an average of about 11% per year (Odd Ratio: 0.89 [0.84, 0.95]) (GLM, Likelihood Ratio test of the model, *p* = 0.0003) (Figure 6C). For co-infections, however, no significant (GLM, Likelihood Ratio test of the model, *p* = 0.5) change in prevalence could be demonstrated between 2005 and 2016 (Odd Ratio: 0.95 [0.81, 1.11]) (Figure 6D).

### *In vitro*-infection of IPL-LD-65Y cells

To explain the obvious success of *N. ceranae* over *N. apis* in the studied honey bee population in summer, we experimentally analyzed the proliferative capacity of both microsporidian species in infected cells at temperatures between 21° and 33°C. To this end, we used an established cell culture model for *N. apis* and *N. ceranae* based on experimentally infecting cultured IPL-LD-65Y-cells. This insect cell line derived from *Lymantria dispar* had been shown to support replication of both microsporidian species (Gisder et al., 2011).

**Figure 7:**
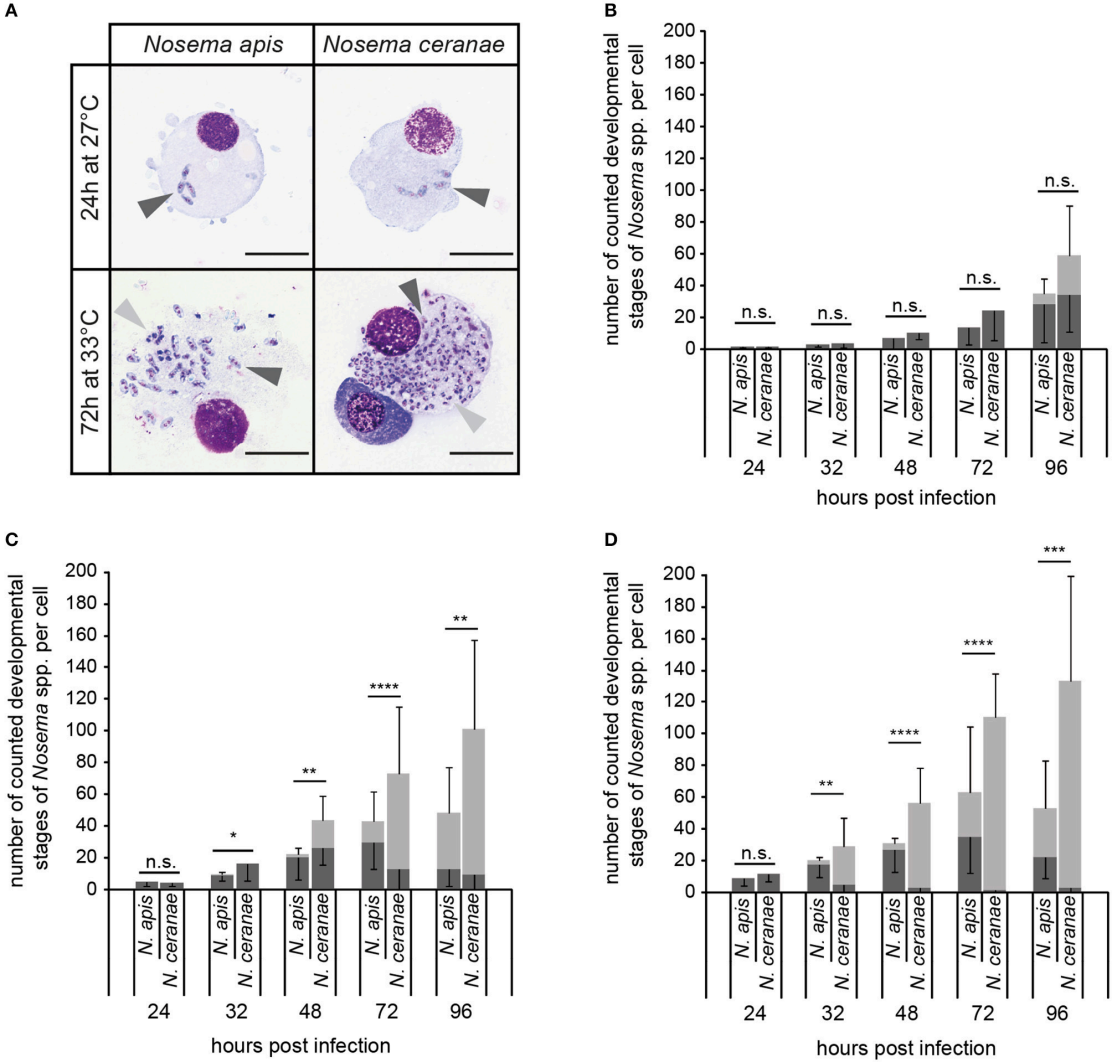
*In vitro*-infection of IPL-LD-65Y cells with *N. apis*- and *N. ceranae*-spores. *Nosema* spp. proliferation was determined by counting the number of developmental stages of merogony (**A**; meronts, dark gray arrow heads) and sporogony (**A**; sporonts and intracellular spores, light gray arrow heads) in infected cells (scale bars = 25 μm) incubated at 21°C **(B)**, 27°C **(C)**, and 33°C **(D)**. Dark gray bars represent the number of meronts per cell (mean of 10 cells ± *SD*), light gray bars represent the number of sporonts/spores per cell (mean of 10 cells ± *SD*). Statistical analysis of the number of developmental stages was performed with student's *t*-tests for each time point and temperature. Statistical results given above the bars refer to the comparison of sporonts/spores produced by *N. apis* and *N. ceranae* (not significantly different: n.s., *p* ≥ 0.05; significantly different: ^*^, 0.05 < *p* < 0.01; ^**^, 0.01 < *p* < 0.001; ^***^, 0.001 > *p* ≥ 0.0001; ^****^ *p* < 0.0001).

Intracellular proliferation of *N. apis* and *N. ceranae* at three different temperatures (21°, 27°, and 33°C) was evaluated by determining the number of the developmental stages per cell produced during merogony (meronts) and sporogony (sporonts/spores) of *Nosema* spp. (Figure 7A). The number of both meronts and sporonts/spores increased for *N. apis* as well as for *N. ceranae* over the observation time period of 96 h at all three tested temperatures. However, the number of the different developmental stages varied between *N. apis* and *N. ceranae* infected cells depending on incubation time and incubation temperature. At 21°C, there was no significant difference in the proliferative capacity of *N. ceranae* and *N. apis* in infected cells for both meronts and sporonts/spores at all tested time points (24, 32, 48, 72, and 96 h post-infection) (all *p* > 0.05) (Figure 7B). However, at 27°C and even more so at 33°C, a higher proliferation rate and a faster proliferation of *N. ceranae* compared to *N. apis* could be observed. In infected cells which were incubated at 27°C (Figure 7C), the number of meronts was not significantly different between *N. apis* and *N. ceranae* after 32 and 72 h post-infection (*p* > 0.05) but was significantly different at time points 24, 48, and 96 h post-infection (*p* > 0.05). More importantly, the number of counted sporonts/spores at 32, 48, 72, and 96 h post-infection was significantly higher (*p* < 0.05) in *N. ceranae*- than in *N. apis*-infected cells (Figure 7C). When the host cells were incubated at 33°C, the number of *N. apis* meronts was significantly (all *p* < 0.01) higher than the number of *N. ceranae* meronts at 32, 48, 72, and 96 h post-infection (Figure 7D) and the numbers of sporonts/spores were significantly higher at 32, 48, 72, and 96 h post-infection (all *p* < 0.01) in *N. ceranae*-infected host cells than in cells infected with *N. apis*.

## Discussion

### Prevalence of *N. ceranae* infections follows the same seasonality as *N. apis* infections

*Nosema ceranae* is an emergent pathogen of the Western honey bee *A. mellifera*. Its first detection in colonies of *A. mellifera* dates back a decade (Higes et al., 2006; Huang et al., 2007), although, it obviously switched host from *A. cerana* to *A. mellifera* about 40 years ago (Teixeira et al., 2013) and is now endemic in the global *A. mellifera* population. Several differences between *N. ceranae* and its congener *N. apis* have been reported, including differences in virulence, in seasonality of infections, and in temperature dependence of spore germination and biotic potential. Most of these differences seem to work in favor for *N. ceranae* resulting in its continous spread in the bee population and a supersession of *N. apis* in many regions (Klee et al., 2007; Chen et al., 2008; Williams et al., 2008; Invernizzi et al., 2009; Chen and Huang, 2010; Stevanovic et al., 2011; Yoshiyama and Kimura, 2011; Copley et al., 2012). However, studies claiming lack of seasonality of *N. ceranae* or replacement of *N. apis* in a given honey bee population are rather short-termed studies rarely performed over more than 2 years and most often involving only a limited set of samples. Our epidemiology data based on observing a cohort 230 bee colonies sampled twice a year over 12 years now revealed a different picture at least for the study area.

*Nosema apis*-infections are known to follow a seasonal pattern with spring prevalence being higher than autumn prevalence. This seasonality can be explained by the pathobiology of *N. apis*: (i) Only the spores of *N. apis* are infective; (ii) older bees are more likely to be infected and carry more spores; and (iii) spores are most efficiently transmitted through the fecal-oral route (Bailey, 1967; Bailey and Ball, 1991). Therefore, *N. apis* transmission within the colony is favored by conditions with low or no brood rearing and forcing adult bees to stay inside the hive for longer periods and to have close in-hive contacts (Bailey, 1967; Bailey and Ball, 1991). These conditions are regularly fulfilled during the winter months in climatic zones with winter temperatures falling below 10°C not allowing bees to fly out (Winston, 1987). Instead, in these regions honey bee colonies hibernate by longlived adult winter bees forming a winter cluster around the queen bee and not leaving the hive for weeks or months until weather conditions allow cleansing and foraging flights and restarting brood rearing to replace the old winter bees (Winston, 1987). This explains why *N. apis*-infection levels increase over winter but normally decrease over summer when the rather shortlived summer bees are engaged in foraging, are able to defecate outside the hive, and when newly raised bees regularly replace older more heavily infected bees (Bailey, 1967; Bailey and Ball, 1991; Retschnig et al., 2017).

In contrast, *N. ceranae*-infections were described to lack this characteristic seasonality (Higes et al., 2006, 2010) suggesting fundamental differences in pathobiology and preferred routes of transmission which would be interesting to investigate. To analyse this suggested lack of seasonality, we collected bee samples in spring and autumn without gap over 12 years from a cohort of around 230 honey bee colonies and analyzed all samples for the presence of *Nosema* spp. spores and performed molecular species differentiation in all *Nosema* spp.-positive samples. Surprisingly, the data clearly disproved that *N. ceranae*-infections differ from *N. apis*-infections in regard to seasonality. Quite the contrary was true: All four infection categories, *Nosema* spp.-, *N. apis*-, *N. ceranae*-, and co-infections, followed the same seasonal pattern with spring prevalence of infection regularly being higher than autumn prevalence suggesting that *N. ceranae* and *N. apis* circulating in Northeast Germany are similar in regard to pathobiology and preferred transmission routes. Since reports on the lack of seasonality predominantly stem from South Europe (Higes et al., 2006, 2010), further experimental studies are necessary to analyse whether the differences in seasonality between the Northern and Southern parts of Europe are due to climatic factors or intraspecies differences in *N. ceranae*.

### No evidence for a general advantage of *N. ceranae* over *N. apis* and for an overall replacement of *N. apis* by *N. ceranae*

In many regions of the world, prevalence data collected for *N. apis* and *N. ceranae* indicated that *N. ceranae* has become the dominant species in the worldwide honey bee populations and it was suggested that *N. ceranae* has replaced or is about to replace its congener globally (Chen et al., 2012; Martin-Hernandez et al., 2012). However, in Europe, a South to North gradient was observed with *N. ceranae* being dominant in Southern European countries already 10 years ago while at that time *N. apis* was still dominant in the Northern part of Europe (Klee et al., 2007) which might reflect an already discussed climatic aspect in *N. ceranae* spread and assertiveness (Fenoy et al., 2009; Martin-Hernandez et al., 2009; Gisder et al., 2010; Chen et al., 2012; Natsopoulou et al., 2015).

Congruent with this South to North gradient (Klee et al., 2007), at the beginning of our epidemiology study we observed very low levels of prevalence for *N. ceranae*-infections in Northeast Germany compared to *N. apis*-infections. This starting condition, the size of the cohort, and the design and duration of the study provided a unique opportunity to follow the spread of the emerging pathogen *N. ceranae* and analyse the impact of this spread on its congener *N. apis*, well established in the observed honey bee population. Our epidemiology data show that starting from a very low level, the prevalence of *N. ceranae*-infections significantly increased continuously in the observed cohort of honey bee colonies during the last 12 years. This increase was true for both time points of sampling, in spring (showing the development over winter) and autumn (showing the development over summer) clearly indicating that *N. ceranae* became successfully established and expanded its presence in the honey bee population of Northeast Germany.

With regard to replacement of *N. apis* by *N. ceranae*, the obtained epidemiology data showed a complex picture. For assuming a replacement process at the population level, *N. apis* infection prevalence should have concomitantly decreased during the study period. However, a significant decrease in *N. apis*-infection prevalence was only observed for autumn indicating that during the bee season in summer *N. ceranae* successfully competed with *N. apis* at the population level over the course of the study. Surprisingly and in contrast to autumn, no significant change in *N. apis* infection prevalence was evident in spring despite a significant increase in *N. ceranae* prevalence. Therefore, no replacement of *N. apis* by *N. ceranae* in the honey bee population of Northeast Germany took place over winter during the last 12 years. Instead, the increase in *N. ceranae* prevalence in spring came on top of the unaltered *N. apis* infection prevalence suggesting that the two microsporidian parasites did not compete with each other over winter at the colony and population level. In addition, the long term stability of *N. apis*-infection frequency in spring indicate, that whatever mechanisms are acting on *N. apis* during summer and causing its decrease in the population, they are compensated for and reversed during winter preventing a supersession of *N. apis* through *N. ceranae* in the observed honey bee population.

Replacement of *N. apis* by *N. ceranae* at the population level during summer but not during winter points to different mechanisms acting on or influencing the two microsporidian parasites in summer and over winter. Although the exact mechanism responsible for presence (summer) and absence (winter) of replacement at the population level still remain elusive, experimental data providing explanations at the individual bee level for the increase in *N. ceranae* infection prevalence over summer exists. In a recent study by Martin-Hernandez et al. (2009), infection experiments with caged bees were performed at different temperatures and the “biotic index” was calculated for both microsporidia as the total *N. apis* or *N. ceranae* spore count per day after infection. This “biotic index” was higher for *N. ceranae* than for *N. apis* at 25°C but no significant difference could be observed at 33°C (Martin-Hernandez et al., 2009). Although these results did not provide convincing proof for an advantage of *N. ceranae* over *N. apis* during summer, they pointed into an interesting direction. Therefore, we extended the approach and performed infection experiments in cell culture (Gisder et al., 2011), which allowed a detailed analysis of the time course of proliferation and of the proliferative potential of *N. ceranae* and *N. apis* at different temperatures. Our *in vitro* results revealed a significant advantage of *N. ceranae* over *N. apis* at 27° and 33°C, the normal range of daily maximum temperatures in summers in Northeast Germany. At both temperatures, *N. ceranae* completed its replicative cycle faster and replicated more efficiently than *N. apis*. These results were in accordance with a recent study, suggesting a generally higher proliferation rate for *N. ceranae* compared to *N. apis* in experimentally infected, caged bees incubated at 30°C for 20 days (Huang and Solter, 2013). Earlier and higher production of spores, which are transmitting the disease within and between colonies, may translate into higher infection prevalence at population level. These data explain an increase of *N. ceranae* infection levels, however, they still do not explain the observed replacement of *N. apis* by *N. ceranae* over summer.

For replacement of *N. apis* by *N. ceranae*, a simple increase in *N. ceranae* infection prevalence is not sufficient but a successful interspecies competition, with *N. ceranae* at least more often than *N. apis* winning the game, is necessary. Again, only experimental data at the individual bee level are available. Co-infection experiments with caged bees and simultaneous feeding of *N. apis* and *N. ceranae* spores did not provide evidence for intra-host competition between the two species (Forsgren and Fries, 2010; Milbrath et al., 2015). In contrast, sequential feeding of spores of the two species resulted in within-host competition: The first parasite significantly inhibited the growth of the second, regardless of species (Natsopoulou et al., 2015). This would have prevented the spread of *N. ceranae* because *N. apis* had been present in the bee population before *N. ceranae* arrived and would always have been first. However, this so-called “priority effect” proved to be asymmetric and *N. ceranae* exhibited a stronger inhibitory effect on *N. apis* than *N. apis* on *N. ceranae* (Natsopoulou et al., 2015). Mathematical modeling proposed that this priority effect will result in a successful replacement process at population level even when taking into account that the cold sensitivity of *N. ceranae* but not of *N. apis* spores (Fenoy et al., 2009; Gisder et al., 2010) provides a disadvantage for *N. ceranae* during cold winters (Natsopoulou et al., 2015).

However, for spring samples our epidemiology data clearly showed that although *N. ceranae*-infection prevalence increased over time, this increase did not result in a replacement of *N. apis*. Remarkably, *N. apis*-infection prevalence in spring remained rather stable over the 12 years study period although the autumn infection prevalence and, hence, the infection prevalence at the beginning of winter, has been declining during this period. Therefore, the two *Nosema* species rather not competed during winter and the mechanisms promoting the increase of *N. ceranae* in the studied honey bee population over winter did not influence the prevalence of *N. apis*.

Furthermore, we observed higher than expected co-infection rates in spring suggesting that there is no interspecies within-host competition at colony or population level during overwintering. The co-infection levels rather suggested that an infection with any one of the two microsporidia pre-existing in a colony favored an additional infection of the colony with the other microsporidium. This is in contrast to the above mentioned report (Natsopoulou et al., 2015) showing interspecies within-host competition with a priority effect favoring the spread *N. ceranae* over *N. apis*. However, this inter-species competition was shown at the individual bee level whereas our epidemiology data concern the colony and population levels. And indeed, at the colony and population level it is hardly conceivable how an *N. ceranae* infection of one bee or colony might inhibit a nestmate or a neighboring colony, respectively, to become infected by *N. apis*- and the other way round. Actually, the concept of interspecies within-host competition of an obligate intracellular parasite due to competition for the same limited cellular energy resources cannot easily be translated to the colony or population level where limitation or shortage of resources (in this case: new hosts) is not yet the problem. However, if the prevalence of *N. ceranae*-infections keeps increasing like it did over the last 12 years, within-colony and between-colony competition might become an issue once all colonies are infected with either one of the microsporidia. Therefore, a continuation of this study will further our understanding of the long term epidemiology and interspecies competition at population level of these two important honey bee pathogens.

## Author contributions

EG and SG conceived and designed the study and the experiments. SG, VS, and LH carried out the experiments and the microscopic and molecular diagnosis of *Nosema* spp. SG, VS, and DG performed the statistical analysis. EG supervised all work, SG supervised the laboratory experiments, and DG supervised the statistical analysis. SG and EG wrote the paper. All authors revised the manuscript and approved the final version.

### Conflict of interest statement

The authors declare that the research was conducted in the absence of any commercial or financial relationships that could be construed as a potential conflict of interest.
